# Development of a Rapid Visual Detection Assay for Duck Tembusu Virus Using RT-LAMP-CRISPR/Cas12a

**DOI:** 10.3390/ani14233439

**Published:** 2024-11-27

**Authors:** Jimin Chen, Dagang Tao, Fan Yang, Chengfu Pan, Xinguo Bao, Shengsong Xie, Ping Gong, Changzhi Zhao, Ruiyi Lin

**Affiliations:** 1 College of Animal Sciences, Fujian Agriculture and Forestry University, Fuzhou 350002, China; jmchen@fafu.edu.cn (J.C.); 12306009008@fafu.edu.cn (F.Y.); 52306009028@fafu.edu.cn (C.P.); 52306009010@fafu.edu.cn (X.B.); 2 Key Laboratory of Agricultural Animal Genetics, Breeding and Reproduction of Ministry of Education & Key Lab of Swine Genetics and Breeding of Ministry of Agriculture and Rural Affairs, Huazhong Agricultural University, Wuhan 430070, China; dgtao@webmail.hzau.edu.cn (D.T.); ssxie@mail.hzau.edu.cn (S.X.); zhaochangzhi@yzwlab.cn (C.Z.); 3 Animal Husbandry and Veterinary Research Institute, Wuhan Academy of Agricultural Sciences, Wuhan 430208, China; gongping09@foxmail.com

**Keywords:** Duck Tembusu virus, Cas12a, RT-LAMP

## Abstract

Duck Tembusu virus (DTMUV) is an emerging flavivirus that has inflicted significant economic losses on China’s poultry industry. Rapid and accurate detection of DTMUV is crucial for effective prevention and control measures. In this study, we developed a novel, rapid visual detection assay that combines reverse transcriptase loop-mediated isothermal amplification (RT-LAMP) with the CRISPR/Cas12a system for on-site detection of DTMUV. Our results demonstrate that this assay can sensitively and specifically detect the specific DNA plasmids containing the DTMUV *NS3* gene within 100 min, with a limit of detection as low as 19.3 copies/μL. We successfully applied the RT-LAMP-CRISPR/Cas12a assay to diagnose DTMUV in eight duck embryos and 11 chicken embryonic fibroblast samples, and the results obtained with direct visualization by the naked eye were consistent with those obtained using real-time RT-PCR. Overall, our RT-LAMP-CRISPR/Cas12a assay is a reliable, sensitive, specific, and user-friendly method that holds great promise for early on-site detection of DTMUV in clinical samples, facilitating timely interventions and improved disease management in the poultry industry.

## Simple Summary

The decline in egg production among duck flocks infected with duck Tembusu virus (DTMUV) has incurred substantial economic losses to affected countries and regions, yet an effective treatment remains elusive. The prevention and control of DTMUV largely depend on early and rapid detection, highlighting the dire need for clinically ideal detection methods specifically designed for DTMUV detection. Despite the availability of multiple detection approaches, we herein develop a visual detection method for DTMUV that integrates the virus nucleic acid amplification technique known as loop-mediated isothermal amplification (LAMP) with the novel technology of the clustered regularly interspaced short palindromic repeats/Cas12a (CRISPR/Cas12a) nuclease system. This visual detection assay is characterized by its rapidness, sensitivity, specificity, and portability, demonstrating the potential for the early clinical diagnosis of DTMUV.

## 1. Introduction

Duck Tembusu virus is a single-stranded positive-sense RNA virus belonging to the group *Ntaya* virus within the genus *Flavivirus* of the family *Flaviviridae* [1,2]. In recent years, DTMUV infections have been reported in poultry in China, Malaysia, Vietnam, and Thailand within Asia [3,4,5,6,7,8]. Notably, a variant strain of DTMUV, named TMUV-HQ22, isolated from diseased geese in Anhui, China, in 2022, demonstrates significant pathogenicity in goslings and ICR mice [9]. Similar to other flaviviruses, DTMUV possesses a positive-sense genomic RNA of approximately 11 kb, consisting of a single open reading frame (ORF) flanked by 5′ and 3′ untranslated regions (UTRs). The ORF of DTMUV encodes three structural proteins, namely capsid protein C, precursor membrane protein prM, and envelope protein E, as well as seven non-structural proteins (NS1, NS2A, NS2B, NS3, NS4A, NS4B, and NS5) [10,11]. The Tembusu virus was first discovered in the mosquito Culex tritaeniorhynchus in 1955 [12]. Between 1955 and 2000, cases of encephalomyelitis and growth retardation in chicks caused by Tembusu virus infection were identified in Malaysia [13]. The first DTMUV outbreaks were reported in breeding ducks in Shanghai and other regions in China in 2010 [14]. DTMUV infection leads to duck Tembusu virus disease characterized by decreased egg production and neurological damage in ducks [15,16]. Infected ducks exhibit symptoms such as anorexia, diarrhea, decreased egg production, and paralysis, with mortality rates ranging from 5% to 30% [14,17,18]. Currently, there is no effective drug treatment for DTMUV infection, and the host range of DTMUV has expanded to include Muscovy ducks, geese, laying hens, pigeons, sparrows, and other avian species [19,20]. The virus spreads rapidly through diverse transmission routes, including direct contact, aerosol transmission, and even vertical transmission among avian populations, posing a serious threat to the healthy development of the poultry industry [21,22]. Therefore, early diagnosis and control of DTMUV infection are crucial for the poultry breeding industry.

Traditional detection methods for duck Tembusu virus (DTMUV) include reverse transcription-polymerase chain reaction (RT-PCR), real-time RT-PCR, reverse transcription loop-mediated isothermal amplification (RT-LAMP), enzyme-linked immunosorbent assay, and virus isolation. Established RT-PCR detection methods primarily target the *E*, *NS3*, and *NS5* genes, while RT-qPCR detection methods primarily target the *E*, *NS1*, and *C* genes [23,24,25,26]. These laboratory-based methods possess inherent efficiency by enabling the simultaneous detection of multiple viruses in a single reaction, thereby conserving both time and sample material. Additionally, the reduction in reagent usage undoubtedly lowers the cost of detection. However, inconsistent amplification efficiency and the complexity of result analysis increase the risk of false-positive or false-negative outcomes. Furthermore, their reliance on laboratory settings limits their application for field testing. Several RT-LAMP methods targeting the *E* or *NS5* genes have been developed for on-site detection of DTMUV [27,28,29]. These methods utilize four or six primers and can complete the detection process within 25 to 60 min at a constant temperature. Although they cannot detect multiple pathogens simultaneously, they offer a shorter detection time compared to RT-PCR and RT-qPCR. However, in terms of detection cost, they are slightly inferior due to the larger amount of primers required. Notably, to avoid the cumbersome and time-consuming process of gel electrophoresis for result interpretation, RT-LAMP assays allow visual determination of detection results by observing the turbidity or fluorescence of the system. Nevertheless, RT-LAMP is also prone to non-specific amplification, making it difficult to distinguish between target and non-target amplification, which increases the risk of false-positive results and renders the outcomes unreliable [30,31].

Current molecular diagnostic tools based on the clustered regularly interspaced short palindromic repeats (CRISPR)/CRISPR-associated protein 12a (Cas12a) system have emerged as a promising novel generation of diagnostic methods. The activated Cas12a enzyme cleaves target double-stranded DNA (dsDNA) in a cis-manner and exhibits extremely high activity in trans-cleaving non-target ssDNA present in flanking regions [32,33]. By leveraging the collateral cleavage activity of the activated Cas12a enzyme, numerous molecular diagnostic tools have been developed that integrate single-stranded DNA fluorescent reporter probes into the CRISPR/Cas12a system. These systems combine with isothermal amplification techniques, such as recombinase polymerase amplification (RPA), rolling circle amplification (RCA), and loop-mediated isothermal amplification (LAMP), to achieve rapid and visual detection of pathogenic microorganisms [34,35,36,37,38]. Recently, rapid visual CRISPR (RAVI-CRISPR) systems have been developed for the high-sensitivity detection of severe acute respiratory syndrome coronavirus 2 (SARS-CoV-2) and African swine fever virus (ASFV) [39]. In addition, a portable CRISPR/Cas12a and -Cas13a multiplex point-of-care detection platform has also been created, achieving 100% sensitivity and specificity for the analysis of CD163 knockout, lactoferrin (LF) knock-in, and wild-type pig samples [40]. However, despite these advancements, there are no reports on the use of RAVI-CRISPR technology for on-site detection of DTMUV. The diagnostic capabilities vary significantly across different detection platforms or methodologies, yet the early detection and control of DTMUV outbreaks are paramount in preventing economic losses in the poultry industry. Consequently, there is an urgent need for a reliable rapid detection method for DTMUV.

To address this need, we have further developed a RAVI-CRISPR method that integrates reverse transcription loop-mediated isothermal amplification (RT-LAMP) with the CRISPR/Cas12a system. This approach specifically targets the highly conserved *NS3* gene of DTMUV, which plays a crucial role in viral replication and is an ideal target for diagnostic applications. By leveraging this gene, our method enables sensitive, specific, portable, and visually observable detection of DTMUV.

## 2. Materials and Methods

### 2.1. Collection of Duck Embryos and DF-1 Passaged Cells

Eight 10-day-old fertile breeder duck eggs, which contained duck embryos with obvious fetal movement and clearly identifiable blood vessels and were free of maternal antibodies against DTMUV, were divided into two groups and inoculated with the DTMUV FQ-C1 strain in their allantoic cavities. Specifically, five eggs in the DTMUV-infected duck embryo group were inoculated with 200 μL of DTMUV diluted three times with PBS solution, with an egg infectious dose 50 (EID_50_) of the original virus at 1 × 10^−4.5^/0.1 mL. In contrast, three eggs in the DTMUV duck embryo control group were inoculated with 200 μL of PBS solution. Additionally, a chicken embryo fibroblast cell line (DF-1 cells) cultured in two 6-well plates was randomly divided into a cell infection group (9 wells) and a cell control group (2 wells). Each well contained a specified number of cells. When the DF-1 cell density reached approximately 90%, the infection group was inoculated with 0.01 tissue culture infectious dose 50 (TCID_50_) of DTMUV FQ-C1 strain, with a TCID_50_ of the virus at 10^−3.8^/0.1 mL, while the control group was inoculated with an equal volume of RPMI 1640 medium (Cat No. 11875119, Gibco, Grand Island, NY, USA). After incubation at 37 °C for 2 h, the medium was replaced with a normal cell culture medium containing 2% serum for continued cultivation. Cell supernatants from the infected group were collected at multiple time points post-infection (6–72 h post-infection), as shown in Table S1. Ultimately, the total RNA from the allantoic fluid of duck embryos and the DF-1 cell supernatants was collected for further investigation.

### 2.2. Nucleic Acid Extractions and Preparations

The genomic RNA of DTMUV was extracted from the allantoic fluid or cell culture supernatant of inoculated duck embryos or cells after 48 h using the Viral RNA/DNA Extraction Kit (Cat No. 9766, TAKARA, Tokyo, Japan). The extracted RNA was then reverse-transcribed into complementary DNA (cDNA) using the NovoScriptR Plus All-in-one 1st Strand cDNA Synthesis SuperMix (E047, Novoprotein, Suzhou, China). The partial DTMUV *NS3* gene fragment of 832 bp (GenBank accession number: KX977555.1) was amplified from the DTMUV cDNA using PCR with *NS3*-PCR-TAclone primers (Table 1). The resulting amplicon was then cloned into the pCE2 TA/Blunt-Zero vector (C601-01, Vazyme, Nanjing, China). Similarly, the partial Muscovy duck reovirus (MDRV) *σC* gene fragment of 810 bp (GenBank accession number: KX831117.1) was synthesized and cloned into the PUC57 vector (Cat No. 54338, addgene, Waterton, MA, USA).

### 2.3. Primers and crRNAs Design

The LAMP primers for specifically amplifying the DTMUV *NS3* gene fragment were designed using PrimerExplorer V5 (https://primerexplorer.jp/e/ (accessed on 5 November 2023)), a tool that allows for the design of specific and efficient primers. PCR primers specific to the DTMUV *NS3* gene fragment were designed using the Primer-BLAST tool available at the National Center for Biotechnology Information (NCBI). For RT-PCR and RT-qPCR primers used to detect DTMUV, we referred to the previously reported literature [41,42]. Additionally, five crRNAs targeting the *NS3* gene of DTMUV were designed using CRISPR-Offinder (http://123.57.239.141:8080/home (accessed on 9 November 2023)) [43], a tool that allows for the design of specific and efficient guide RNAs. The criteria used to design these crRNAs included their specificity, efficiency, and off-target effects. The designed crRNAs are listed in Table S2.

### 2.4. Preparation of crRNAs

To amplify the crRNA template directly through PCR, forward primers containing the T7 promoter and reverse primers targeting the crRNA sequence were used. The reaction conditions for this PCR used to construct crRNA are as follows: initial denaturation at 95 °C for 3 min; followed by 30 cycles of denaturation at 95 °C for 15 s, annealing at 60 °C for 15 s, and extension at 72 °C for 10 s; with a final extension step at 72 °C for 5 min. The 3′ ends of the forward and reverse primers possessed a complementary 15-base pair overlapping sequence to facilitate efficient amplification. The resulting PCR amplicons were then purified from agarose gels using the FastPure Gel DNA Extraction Mini Kit (DC301-01, Vazyme, Nanjing, China). The purification efficiency was assessed by measuring the concentration and purity of the eluted DNA on a spectrophotometer, ensuring that the A260/A280 ratio was within an acceptable range (typically 1.8–2.0) to indicate high-quality, purified DNA. Utilizing the purified product as the DNA template, in vitro transcription was performed with the HiScribe T7 High Yield RNA Synthesis Kit (E2040S, NEB, Beverly, MA, USA). To eliminate residual DNA, DNase I (EN401, Vazyme, Nanjing, China) was added to the transcription products and incubated at 37 °C for 10 min. Finally, the RNA transcripts were extracted with phenol/chloroform and precipitated to obtain pure crRNA products. The integrity of the RNA was verified by electrophoresis on a 4% agarose gel, which should display a distinct and sharp band indicative of intact RNA. Additionally, the concentration of the transcribed RNA was measured using a spectrophotometer, and the A260/A280 ratio was checked to ensure it fell within the expected range for RNA, typically between 2.0 and 2.2.

### 2.5. Workflow of the RT-LAMP-CRISPR/Cas12a Assay

The workflow of this method is as follows: (1) Rapid RNA Extraction: Duck embryo allantoic fluid or cell samples were used for rapid RNA extraction within 20 min. (2) RT-LAMP: The extracted RNA underwent RT-LAMP isothermal amplification, generating double-stranded target DNA after a 40-min reaction at 65 °C. (3) Cas12a cleavage assay: Under the guidance of specific crRNA, the Cas12a enzyme cleaved for 15 min at 37 °C. The activated Cas12a enzyme exhibited its non-selective collateral cleavage activity toward ssDNA. (4) Fluorescent Detection: The fluorescent ssDNA reporter was labeled with a 5′ROX fluorophore reporter and a 3′BHQ2 quencher. This combination enabled the visual detection of DTMUV RNA within approximately 80 min. The fluorescence signal is generated through the collateral cleavage activity of Cas12a. (5) Visual Detection: The orange fluorescence produced by positive samples was detectable by the naked eye under a simple blue light transilluminator.

### 2.6. Reverse Transcription PCR (RT-PCR) Assay

Using the DTMUV cDNA as a template, the reaction was primed with NS5-PCR-F and NS5-PCR-R primers [42], which were specifically designed to target the *NS3* gene. The target sequence was amplified by PCR using 2 × Taq Master Mix (P111-01, Vazyme, Nanjing, China) under optimized conditions. The RT-PCR products were analyzed by agarose gel electrophoresis, and the presence or absence of the expected amplicon (~211 bp) was used to determine whether the result was positive or negative, respectively.

### 2.7. Quantitative Reverse Transcription PCR (RT-qPCR) Assay

The DTMUV *NS3* gene was quantified using real-time quantitative PCR (RT-qPCR) to determine the viral nucleic acid copy concentration. The NovoStart^®^ SYBR qPCR SuperMix Plus Kit (E096-01A, novoprotein, Suzhou, China) was used for this purpose. The RT-qPCR reaction mixture consisted of 20 μL, comprising 10 μL of 2×NovoStart^®^ SYBR qPCR SuperMix Plus, 1 μL each of 10 μM forward and reverse primers, 2 μL of cDNA template, and 6 μL of DEPC-treated water. The reaction protocol was set as follows: initial denaturation at 95 °C for 1 min, followed by 45 cycles of 95 °C for 20 s, 60 °C for 20 s, and 72 °C for 30 s. The entire reaction was conducted in a CFX96 Touch Real-Time PCR Detection System (Bio-Rad, Hercules, CA, USA). To establish a standard curve for copy number quantification, a series of 10-fold dilutions of the DTMUV *NS3* cloned plasmid were used, ranging from 10^2^ to 10^7^ copies per reaction. Subsequently, the mean threshold cycle (CT) values for each group were determined, and the CT values of individual samples were interpolated into the standard curve to accurately calculate the viral nucleic acid copy concentration. The detection limit was set at a CT value of 35, where samples with CT values above this threshold were considered negative for DTMUV *NS3* gene amplification, while those with CT values below 35 were deemed positive.

### 2.8. RT-LAMP-CRISPR/Cas12a Assay

The RT-LAMP-CRISPR/Cas12a assay was used for the detection of DTMUV genetic material. The RT-LAMP isothermal amplification reaction mixture consisted of 25 μL, comprising 2.5 μL of 10× isothermal amplification buffer, 1 μL of 8U Bst II DNA Polymerase Large Fragment (P702-01, Vazyme, Nanjing, China), 1.4 mM dNTP mix, 6 mM MgSO_4_, 1.6 μM of each FIP/BIP primer, 0.2 μM of each F3/B3 primer, and 0.8 μM of each LF/LB primer. The reaction was conducted at 65 °C for 40 min on a metal bath. The reaction time and temperature were optimized based on previous experimental results, achieving the highest amplification efficiency and lowest background signal within 40 min at 65 °C, when compared to other tested durations such as 20 min, 30 min, 40 min, and 60 min at the same temperature. Notably, these conditions also align with the recommended reaction conditions specified in the LAMP kit. Subsequently, 2 μL of the LAMP amplification product was analyzed on a 2% agarose gel. The digestion reaction mixture consisted of 20 μL, comprising 0.5 μM of srCas12a-18 (Cat No. SRB-CAS-001, ShangRuiBio, Wuhan, China), 2 μL of 10× CutSmart buffer (B6004, NEB, Beverly, MA, USA), 1 μM crRNA, and 20 μM ssDNA-FQ reporter (5′-ROX-N12-BHQ2-3′). The reaction was incubated at 37 °C for 15 min and then at 98 °C for 2 min. These incubation times were chosen to balance cutting efficiency and background signal, with 15 min at 37 °C yielding the optimal results. Following the reaction, the tubes were transferred to a dark surface, where the fluorescence signal was visually inspected under blue light and UV illumination.

### 2.9. RT-LAMP-CRISPR/Cas12a Assay Sensitivity and Specificity Assessment

The sensitivity and specificity of the RT-LAMP-CRISPR/Cas12a assay were evaluated to determine its potential for detecting DTMUV genetic material. The sensitivity of the RT-LAMP-CRISPR/Cas12a assay was evaluated by serially diluting cloned plasmids containing the DTMUV *NS3* gene to concentrations ranging from 1.93 × 10^4^ to 1.93 × 10^−1^ copies/μL. The diluted plasmids were then amplified using RT-LAMP with DTMUV *NS3* gene-specific primers. The specificity of the RT-LAMP-CRISPR/Cas12a assay was evaluated by testing both DTMUV *NS3* cloned plasmids (positive control) and MDRV *σC* synthetic plasmids (negative control) using the RT-LAMP-CRISPR/Cas12a assay. The resulting RT-LAMP products were subsequently digested using a CRISPR/Cas12a reaction containing crRNA specifically targeting the DTMUV *NS3* gene.

### 2.10. Statistical Analysis

The sensitivity and specificity of the RT-LAMP-CRISPR/Cas12a assay were further evaluated by analyzing the fluorescence intensity values. Fluorescence intensity values were analyzed using GraphPad Prism 9.5 software (La Jolla, CA, USA), and the mean ± standard error of the mean (SEM) was calculated to quantify the variability in fluorescence signal.

## 3. Results

### 3.1. Establishment of RT-LAMP-CRISPR/Cas12a Assay for Detection of DTMUV

**Figure 1:**
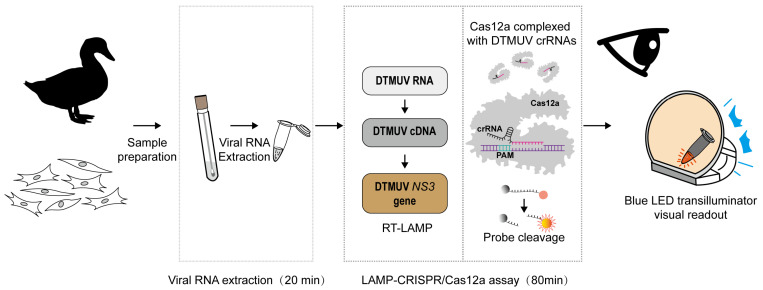
Schematic diagram of the RT-LAMP-CRISPR/Cas12a assay workflow.

DTMUV infection is a significant threat to duck farming, and rapid diagnosis is essential for controlling outbreaks. Here, we developed an RT-LAMP-CRISPR/Cas12a assay to detect DTMUV RNA. The detection assay comprises the following stages: (1) RNA is rapidly extracted from known infected samples, a process that takes approximately 20 min. (2) The extracted RNA is subsequently subjected to RT-LAMP for about 40 min, followed by a trans-cleavage reaction with Cas12a enzyme for an additional 15 min. (3) Finally, the detection results are directly visualized under a blue light gel cutter, where a clear orange fluorescence indicates a positive result, and the absence of such fluorescence indicates a negative result. It is noteworthy that the entire detection process takes only approximately 100 min (Figure 1).

### 3.2. Screening Optimal Prime Pairs and crRNAs for RT-LAMP-CRISPR/Cas12a Assay for DTMUV Detection

**Figure 2:**
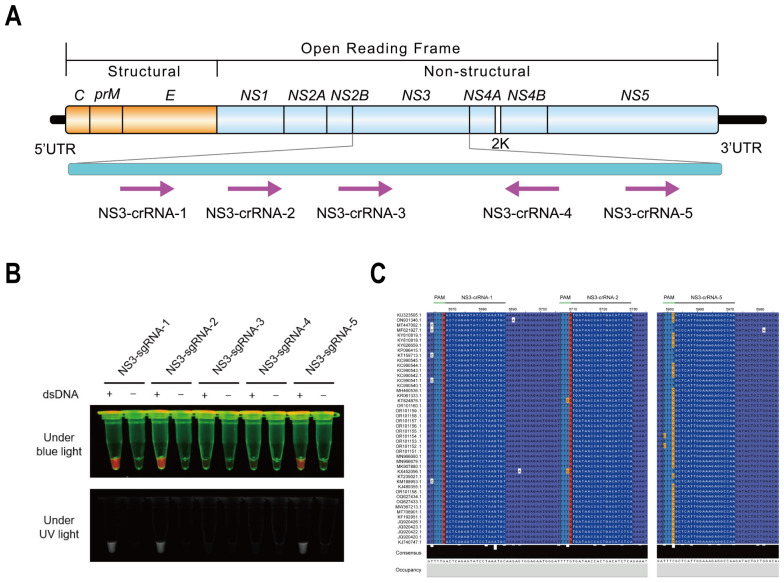
Validation of the highly active crRNA for RT-LAMP-CRISPR/Cas12a detection of DTMUV. (**A**) Schematic diagram of the genome organization of DTMUV showing the *NS3* gene (cyan) with the corresponding crRNAs (pink). (**B**) Identification of highly active crRNAs targeting the *NS3* gene of DTMUV. dsDNA represents a cloning plasmid template containing a fragment of the *NS3* gene. (**C**) Conservation analysis of highly active crRNAs targeting the *NS3* gene of DTMUV. PAM, protospacer adjacent motif. Capturing images using a smartphone camera in the absence of blue light (470 Nm) and ultraviolet light. UTR, untranslated region; crRNA, CRISPR RNA.

To identify the most effective primer pairs for RT-LAMP and guide RNA (crRNA) for the CRISPR/Cas12a assay, we screened multiple primer pair candidates and crRNA candidates targeting the highly conserved *NS3* gene of DTMUV. Additionally, we utilized a ROX dye ssDNA fluorophore quencher (ssDNA-FQ) reporter, as previously reported [39]. We constructed plasmids containing the DTMUV *NS3* gene using TA cloning and designed a set of LAMP primer pairs and five crRNAs targeting the *NS3* gene (Table 1, Figure 2A). The results showed that both primer pairs and crRNAs targeting different regions of the *NS3* gene exhibited varying detection activities under blue and ultraviolet light (Figure 2B). Specifically, NS3-crRNA-1, NS3-crRNA-2, and NS3-crRNA-5 displayed high detection activities for positive plasmids containing the *NS3* gene and non-template controls. Furthermore, a conservation analysis of the DTMUV target sequences of the three high-activity crRNAs revealed that NS3-crRNA-2 was highly conserved among 76 distinct DTMUV strains recorded in NCBI (Figure 2C, detailed results are presented in Supplementary Figure S1). Consequently, LAMP primer pairs and NS3-crRNA-2 were selected as the highly active primer pair and conserved crRNA for the RT-LAMP-CRISPR/Cas12a detection assay. This optimized assay is suitable for the efficient detection of conserved DTMUV genotypes.

### 3.3. Sensitivity Evaluation of the RT-LAMP-CRISPR/Cas12a Assay for DTMUV Detection

**Figure 3:**
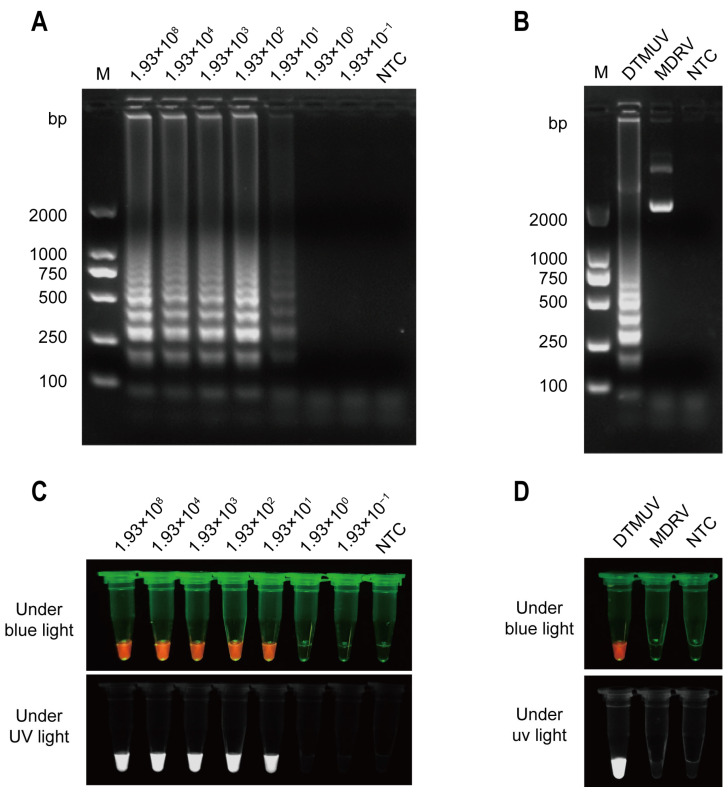
Sensitivity and specificity of the RT-LAMP-CRISPR/Cas12a assay for DTMUV *NS3* gene detection. (**A**) Agarose gel electrophoresis to determine the detection limit of RT-LAMP amplification of the DTMUV *NS3* gene (copies/μL). (**B**) Agarose gel electrophoresis for detection of specific RT-LAMP amplicons. (**C**) Colorimetric signal detection of the 10-fold serial dilutions of DTMUV *NS3* gene (copies/μL) using the LAMP-CRISPR/Cas12a assay. (**D**) Colorimetric signal detection of DTMUV and MDRV using the LAMP-CRISPR/Cas12a assay. Capturing images using a smartphone camera in the absence of blue light (470 Nm) and ultraviolet light. NTC, no template control.

To assess the sensitivity of the RT-LAMP-CRISPR/Cas12a detection method, we constructed a partial *NS3* gene sequence (832 bp) from the cDNA of the DTMUV FQ-C1 strain (GenBank accession number: KX977555.1) using TA cloning into the pCE2 TA/Blunt-Zero plasmid. The cloned plasmid DNA containing the *NS3* gene served as a template. We then performed technical replicates (n = 3) for each condition to ensure the reliability of the results. Using a 10-fold dilution concentration gradient of the *NS3* template, we found that the detection limit of RT-LAMP amplification was 19.3 copies (Figure 3A). This indicates the minimum number of target molecules required for successful detection. The RT-LAMP amplification products were then cleaved using the CRISPR/Cas12a system, followed by colorimetric signal detection. For each dilution, we again performed technical replicates (n = 3) and calculated the mean and standard deviation of the detection sensitivity. The results showed that the detection sensitivity also reached 19.3 total copies (Figure 3C). These findings demonstrate that the RT-LAMP-CRISPR/Cas12a assay can specifically detect DTMUV with high sensitivity.

### 3.4. Specificity of the RT-LAMP-CRISPR/Cas12a Assay for DTMUV Detection

Multiple viral infections or mixed infections, including DTMUV, pose a significant threat to the healthy development of the intensive poultry industry [25,44]. To investigate whether our newly developed method for detecting DTMUV exhibits cross-reactivity with other avian viruses, we evaluated the specificity of the RT-LAMP-CRISPR/Cas12a assay. We used a plasmid synthesized using the *σC* partial gene sequence of Muscovy duck reovirus (MDRV) strain YB as a template to assess the specificity of the RT-LAMP-CRISPR/Cas12a assay for detecting DTMUV. The results confirmed that only the DTMUV-positive plasmid samples produced the expected ladder-like LAMP bands, while the MDRV samples only exhibited the characteristic three bands of the plasmid, namely the supercoiled band, linear plasmid band, and open circular plasmid band (Figure 3B). The large sample loading volume of the MDRV plasmid may have contributed to this result. In contrast, DTMUV-positive plasmid samples did not display these three characteristic bands after undergoing isothermal amplification using LAMP. Furthermore, cleavage of the LAMP products by Cas12a only produced fluorescence in the DTMUV *NS3* gene-derived sample (Figure 3D). This indicates that the RT-LAMP-CRISPR/Cas12a assay for detecting DTMUV exhibits strict specificity, with no cross-reactivity observed with MDRV.

### 3.5. Naked-Eye RT-LAMP-CRISPR/Cas12a Assay for Point-of-Care Testing of DTMUV

**Figure 4:**
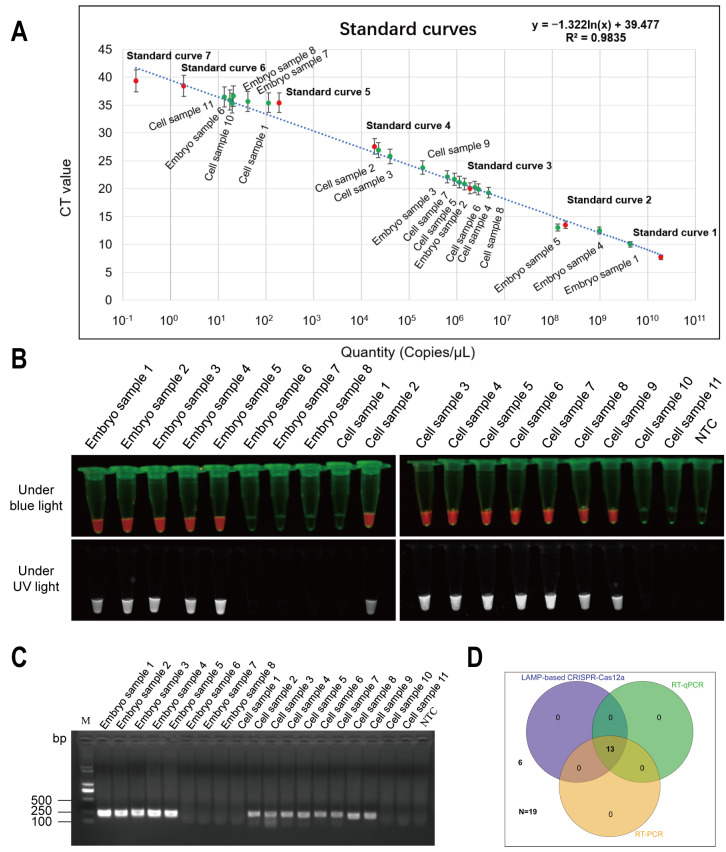
Accuracy of the LAMP-CRISPR/Cas12a assay for DTMUV *NS3* gene detection. (**A**) Standard curve of 10-fold serial dilutions of DTMUV *NS3* gene and copy number quantification of 19 DTMUV-infected or uninfected samples. (**B**) Visual detection of the DTMUV *NS3* gene in test samples using the naked-eye LAMP-CRISPR/Cas12a assay. (**C**) Agarose gel electrophoresis of RT-PCR products from 19 DTMUV-infected or uninfected samples. (**D**) Comparison of the detection results of clinical specimens using RT-qPCR, RT-PCR, and LAMP-CRISPR/Cas12a assays. Capturing images using a smartphone camera in the absence of blue light (470 Nm) and ultraviolet light. NTC, no template control; CT, cycle threshold; Embryo samples 1–5: Urine sac fluid from duck embryos infected with DTMUV; Embryo samples 6–8: Urine sac fluid from uninfected duck embryos; Cell samples 1–9: Supernatant from DF-1 cells infected with DTMUV; Cell samples 10–11: Supernatant from uninfected DF-1 cells.

To validate the accuracy of the RT-LAMP-CRISPR/Cas12a assay, we tested clinical samples and simulated clinical samples, comparing them with existing conventional diagnostic methods. Specifically, to evaluate the ability of the RT-LAMP-CRISPR/Cas12a assay to detect DTMUV in duck embryos and cells, we collected samples from DTMUV-infected duck embryos and chicken embryo fibroblast cell line (DF-1 cells). Subsequently, we performed RT-qPCR analyses. We defined Ct values >35 as negative samples based on the RT-qPCR standard curve of 10-fold diluted DTMUV positive plasmids (Figure 4A). The results showed that allantoic fluid samples from duck embryos No. 1–No. 5 and DF-1 cell samples No. 2–No. 9 could be sensitively detected using RT-qPCR analysis (Supplementary Table S3). Comparing the RT-LAMP-CRISPR/Cas12a assay with RT-qPCR, we found that the results of this method were consistent with RT-qPCR, except for the 6 hpi DF-1 cell infection group, negative control group, and no template control group, which did not detect DTMUV nucleic acid. DTMUV nucleic acid was detected in samples from both duck embryo and DF-1 cell infection groups (Figure 4B).

Additionally, the RT-PCR results were also consistent with the RT-LAMP-CRISPR/Cas12a assay (Figure 4C). The concordance rate of the three detection methods for duck embryo and DF-1 cell samples was 100% (Figure 4D), indicating that the RT-LAMP-CRISPR/Cas12a assay could identify all DTMUV-infected duck embryo and DF-1 cell samples. In addition, its detection accuracy was comparable to RT-qPCR. Moreover, the RT-LAMP-CRISPR/Cas12a assay only requires a portable small thermostat metal bath for amplification and incubation of suspected nucleic acids, facilitating affordable on-site visual detection of DTMUV.

## 4. Discussion

DTMUV, an RNA virus with the potential for zoonotic transmission, poses a threat to poultry farming and human public health safety, as it may undergo mutations detrimental to both under certain environmental selection pressures [45,46,47]. Despite the widespread application of DTMUV vaccines in poultry farms, sporadic infections of DTMUV continue to occur in many regions, posing a risk of outbreaks [5,48]. Consequently, early, rapid, and accurate diagnostic strategies for DTMUV are crucial for disease prevention and timely control. Herein, we have developed a rapid and visual nucleic acid detection assay, integrating RT-LAMP with the CRISPR/Cas12a system, specifically tailored for detecting DTMUV in clinical field samples.

The RT-LAMP-CRISPR/Cas12a assay’s outcome is manifested by a fluorescence visible to the naked eye under a simple blue light transilluminator, facilitated by our meticulously selected highly efficient and active crRNA, a set of highly specific LAMP primers, and a 5′-ROX dye ssDNA-FQ reporter renowned for its exceptional colorimetric sensor properties. Remarkably, the RT-LAMP-CRISPR/Cas12a assay demonstrates a sensitivity threshold of 19.3 total copies per microliter for DTMUV nucleic acids, enabling specific discrimination between DTMUV and MDRV nucleic acids. Furthermore, when tested on eight duck embryos and 11 chicken embryo fibroblast samples, both infected and mock infected, the diagnostic results perfectly aligned with those obtained using conventional RT-PCR and RT-qPCR, achieving a 100% concordance rate. On the other hand, the RT-LAMP-CRISPR/Cas12a detection method unexpectedly surpasses existing detection methods in terms of cost, time, and technical expertise requirements, despite being a qualitative technique that does not provide quantitative detection. First, this analysis requires only basic laboratory equipment, such as a blue light gel cutter costing approximately USD 700, LAMP reagents costing USD 130, and Cas12a enzyme costing USD 105. When combined with labor costs, it can be achieved with only USD 2 per reaction, which means lower operating costs. This is in contrast to traditional DTMUV nucleic acid detection methods, which typically require high-end equipment like PCR machines costing around USD 14,000 and gel imaging systems priced at approximately USD 1400 [25]. Second, the RT-LAMP-CRISPR/Cas12a assay, leveraging isothermal amplification and rapid target recognition, can provide results within 100 min, outpacing RT-PCR and RT-qPCR, which require complex and prolonged reaction conditions [49]. This speed advantage is particularly valuable in situations requiring rapid diagnostic decisions. Lastly, the RT-LAMP-CRISPR/Cas12a assay is designed to be user-friendly, requiring no highly specialized personnel. Visual detection results can be obtained simply by adding the sample and maintaining it at a constant temperature. This makes it more accessible to a wider range of users, including those in remote or underdeveloped agricultural areas where specialized knowledge may be scarce. It is worth emphasizing that regardless of whether the assay is conducted on-site or in a laboratory, the rapid availability of results is crucial for timely poultry management and infection control.

The collateral cleavage activity of Cas12a toward ssDNA upon formation of a ternary complex with crRNA and the target sequence has garnered significant attention and utilization among researchers, as this cleavage capability holds immense potential for the development of next-generation nucleic acid detection technologies [32,50]. Similarly, our established RT-LAMP-CRISPR/Cas12a assay harnesses this enzymatic activity, where during DTMUV nucleic acid detection, the specifically targeted *NS3* gene amplified via LAMP isothermal amplification binds to the crRNA and is subsequently cleaved by the activated Cas12a. Concurrently, Cas12a cleaves the reporter gene, resulting in the emission of a visible orange fluorescence. Prior to this, various DTMUV nucleic acid detection methods have been developed, such as RT-PCR assays [24,51], TaqMan-based real-time PCR [25,26,49], nano-RT-PCR [52], multiplex digital PCR [53], RT-LAMP [42,54,55], and UDG-rRT-LAMP [27]. Notably, RT-PCR and RT-qPCR assays are the two most frequently utilized methods for DTMUV diagnosis; however, among these, only RT-LAMP incorporates fluorescent signals for naked-eye visualization of detection results. Compared to our ROX dye ssDNA-FQ reporter based on CRISPR/Cas12a collateral cleavage, directly adding green fluorescent dyes to the RT-LAMP reaction system to indicate DTMUV detection results is more costly and exhibits inferior fluorescent colorimetry.

Although the fluorescent colorimetry of detection results partially depends on the amount of fluorescent reporter added, the activated Cas12a enzyme sensitively cleaves the fluorescent reporter, minimizing the waste of fluorescent probes in the reaction system, thereby rendering the RT-LAMP-CRISPR/Cas12a assay affordable. Nonetheless, the RT-LAMP-CRISPR/Cas12a assay also confronts the challenge of non-specific amplification during LAMP isothermal amplification, which can lead to false positives in detection results [56,57]. This necessitates rigorous disinfection, personal protection, and the inclusion of negative and blank controls during on-site nucleic acid detection. Generally, RT-PCR and RT-qPCR play pivotal roles in DTMUV nucleic acid detection, but both require lengthy detection processes, such as gel electrophoresis or real-time fluorescence detection, to interpret results. In contrast, our RT-LAMP-CRISPR/Cas12a assay, a CRISPR-based nucleic acid detection method, requires approximately 100 min from DTMUV RNA extraction to fluorescence visualization, and its cost is significantly lower than RT-qPCR technology. The sensitivity and specificity of nucleic acid detection methods are crucial in diagnosing DTMUV infections. The RT-LAMP-CRISPR/Cas12a assay demonstrates high sensitivity with a detection limit of 19.3 copies/μL for DTMUV NS3 nucleic acid, outperforming several other methods [44,58,59,60]. However, the RPA-CRISPR/Cas13a assay has achieved an even higher sensitivity of 1 copy/μL [61].

The RT-LAMP-CRISPR/Cas12a assay also exhibits excellent specificity and accuracy in differentiating between DTMUV-infected and uninfected samples. Nevertheless, its specificity could be further evaluated by screening a broader panel of cloned plasmids or clinical samples containing specific nucleic acids from other viruses that affect ducks. The major advantage of the RT-LAMP-CRISPR/Cas12a assay is its portability and simplicity, requiring minimal equipment, making it an ideal choice for remote farming regions with limited resources. However, several limitations and considerations need to be addressed: (1) Potential false positives or false negatives remain risks of the RT-LAMP-CRISPR/Cas12a assay that must be confronted and addressed. False positives may arise due to cross-reactivity with other viruses or contaminants present in the samples. To mitigate this situation, rigorous validation using a variety of cloned plasmids of duck viruses and clinical samples is necessary. Conversely, false negatives may occur if inhibitors that interfere with the LAMP reaction are present in the samples, or if the viral load is below the detection limit. Therefore, optimizing the sample preparation protocol and ensuring sufficient viral load in the samples is crucial. (2) Adequate clinical sample preparation is a pivotal step in the RT-LAMP-CRISPR/Cas12a assay, as improper handling can lead to inaccurate results. Low-quality RNA is prone to degradation or contamination, while inhibitors potentially present in the samples may interfere with the LAMP reaction. To address these challenges, standardized protocols for sample collection, storage, and extraction should be established. Furthermore, incorporating RNA purification steps and utilizing LAMP enzymes resistant to inhibitors can further enhance the robustness of the assay. (3) Despite the portability and simplicity of the RT-LAMP-CRISPR/Cas12a detection method, it still faces challenges in field implementation, primarily including the risk of cross-contamination between samples during sample collection, reagent and equipment handling under varying environmental conditions by testing personnel, as well as the logistical challenges of transporting samples from remote agricultural areas. To overcome these obstacles, training programs and user-friendly protocols should be established, and alternative validation methods should be provided for situations with limited resources. Furthermore, we have proposed a troubleshooting guide that suggests enhancing the stability of reagents under various conditions through the use of dried or lyophilized reagents, thereby improving the feasibility of field deployment and addressing issues of improper sample handling. For clinical samples that may contain multiple different pathogens, the specificity and accuracy of RT-LAMP-CRISPR/Cas12a detection can be further assessed by comparing its detection results with those of other traditional methods. This approach also addresses the types of false positive and false negative failures. The rational use of clinical samples and bioinformatics tools can compensate for the lack of alternative validation methods in resource-limited environments. Moreover, designing specific primers and crRNA for validation against other related viruses using bioinformatics tools can reduce costs and time. Meanwhile, there is still room for optimization of RT-LAMP-CRISPR/Cas12a detection to further enhance its functionality. First, improving the LAMP primer set and screening for crRNA with enhanced activity can elevate the detection sensitivity. Reasonable gradients in terms of temperature, time, and primer concentration should be set up to screen for the optimal combination. Second, further exploration and optimization are required in the collection, storage, and extraction of poultry clinical samples, as well as in avoiding cross-contamination and ensuring reagent quality. In addition, we emphasize once again the importance of assessing the specificity of the RT-LAMP-CRISPR/Cas12a assay by screening a broader range of cloned plasmids or clinical samples containing specific nucleic acids from other viruses affecting ducks, such as novel duck parvovirus (NDPV), duck plague virus (DPV), and goose astrovirus (GoAstV). Lastly, the RT-LAMP-CRISPR/Cas12a assay should be compared with other diagnostic methods in real-world settings to further validate its accuracy and reliability. Overall, the RT-LAMP-CRISPR/Cas12a assay shows promise as a rapid, accurate, and portable diagnostic tool for DTMUV infections, particularly in resource-constrained remote farming regions.

## 5. Conclusions

This study has successfully developed a novel and robust RT-LAMP-CRISPR/Cas12a system for the detection of DTMUV nucleic acids. This innovative approach offers unparalleled advantages, including portability, reliability, easy visualization, exceptional specificity, and unprecedented sensitivity, all while requiring minimal equipment. These attributes make it an ideal candidate for early, on-site detection of DTMUV, particularly in resource-limited and impoverished poultry farming settings where access to advanced diagnostic facilities is often restricted. The RT-LAMP-CRISPR/Cas12a system has the potential to revolutionize the diagnosis of DTMUV infections, enabling timely interventions and improving disease management in these underserved areas.

## Figures and Tables

**Table 1:** Oligo sequences used in the studies.

Name	Sequences (5′-3′)	Application
*NS5*-PCR-F	GTCATGGATGTCATCTCGCG	RT-PCR
*NS5*-PCR-R	GCTGACAACCTGTTCTCTCC	RT-PCR
*NS3*-PCR-TAclone-F	GAGGCTCACTTCACAGACCC	PCR
*NS3*-PCR-TAclone-R	ACCGCCGGTCATTGTAACTT	PCR
*NS3*-qPCR-F	TTCATGACAGCCACACCTCC	RT-qPCR
*NS3*-qPCR-R	GTCAAGCACACGGCAATCTC	RT-qPCR
DTMUV-*NS3*-LF	GGATACTCTGAGTCAAAACTC	LAMP
DTMUV-*NS3*-LB	AGCGCAGCGGGTCATAGATA	LAMP
DTMUV-*NS3*-F3	GAGATTGCCGTGTGCTTGAC	LAMP
DTMUV-*NS3*-B3	GCACACTTCCTTCTCCATCCTC	LAMP
DTMUV-*NS3*-FIP	CCCATTCTCCACTTTTGCACCCGGCAAGAAGGTAATTCAG	LAMP
DTMUV-*NS3*-BIP	GAAATGGGAGCGAACTTTGGCACTGGTTTAATGCACTTCCG	LAMP
T7-scaffold-crRNA-F	**TAATACGACTCACTATAGGG**TAATTTCTACTAAGTGTAGAT ^1^	In vitro crRNAs transcription
*NS3*-crRNA-1-R	GCACTTAGGATACTCTGAGTATCTACACTTAGTAG	In vitro crRNAs transcription
*NS3*-crRNA-2-R	TGAGATGTCAGTGGTTATCAATCTACACTTAGTAG	In vitro crRNAs transcription
*NS3*-crRNA-3-R	ATCTATGACCCGCTGCGCTCATCTACACTTAGTAG	In vitro crRNAs transcription
*NS3*-crRNA-4-R	GGGTTGGAAGGGATGTGACAATCTACACTTAGTAG	In vitro crRNAs transcription
*NS3*-crRNA-5-R	TTGGCCTCTTTCCAATGAGCATCTACACTTAGTAG	In vitro crRNAs transcription

^1^ The blue-colored base sequence represents the T7 promoter sequence.

## Data Availability

Data are contained within the article and Supplementary Materials.

## Supplementary Materials

The following supporting information can be downloaded at: https://www.mdpi.com/article/10.3390/ani14233439/s1, Supplementary Figure S1: Comparative sequence analysis of LAMP-Cas12a assay amplified regions of DTMUV strains of different genotypes using Jalview; Supplementary Table S1: Grouping and processing of duck embryo and DF-1 cell samples for scientific research purposes; Supplementary Table S2: The sequences of five crRNAs designed by CRISPR-Offinder specifically targeting the *NS3* gene of DTMUV; Supplementary Table S3: Standard curve for 10-fold serial dilution and copy number quantification of the DTMUV *NS3* gene for 19 DTMUV-infected or uninfected samples.
