# Supplementary material for: Development of a Rapid Visual Detection Assay for Duck Tembusu Virus Using RT-LAMP-CRISPR/Cas12a
Source: Animals (Basel). 2024 Nov 27;14(23):3439. doi: 10.3390/ani14233439 (PMC11640670; doi:10.3390/ani14233439)
Supplement: Supplementary file 1 [file animals-14-03439-s001.zip › animals-3310664-supplementary.pdf]

Supporting information

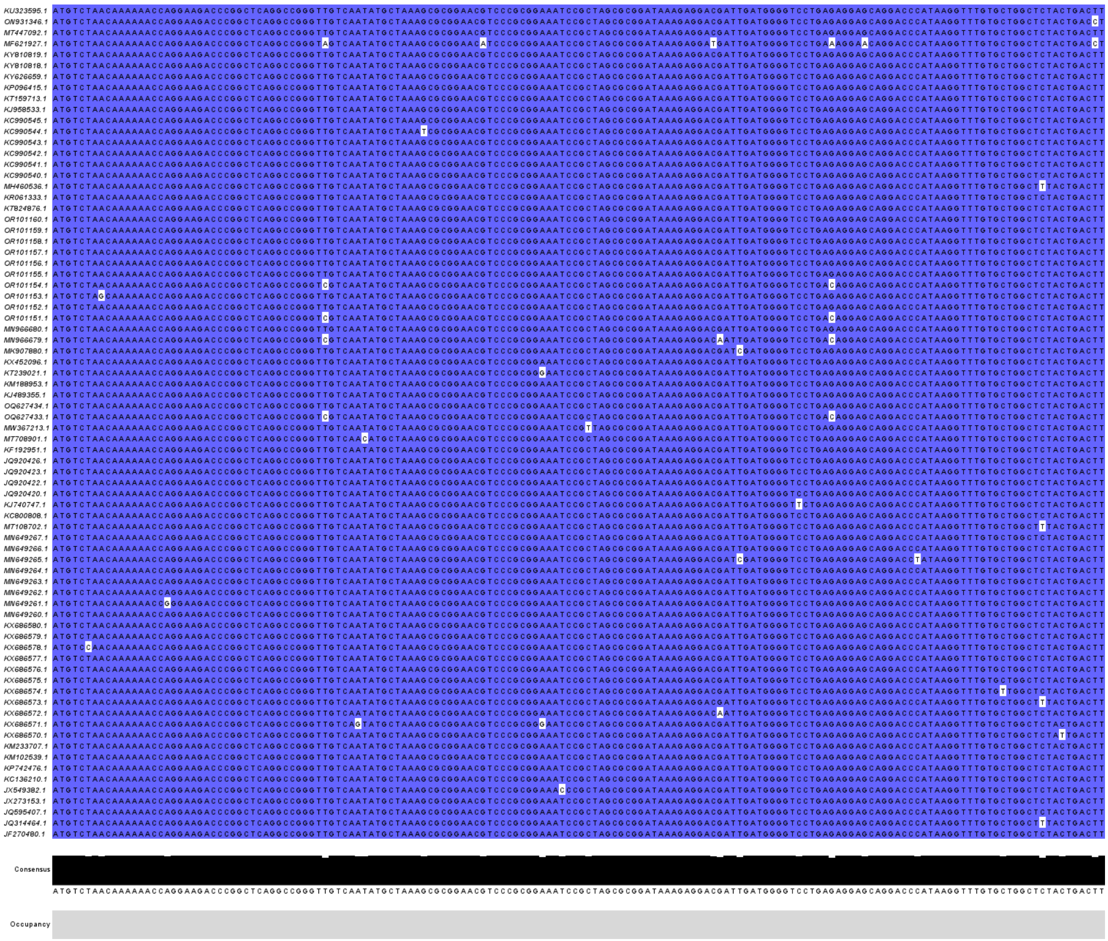

Supplementary Figure S1. Comparative sequence analysis of LAMP-Cas12a assay amplified regions of DTMUV strains of different genotypes by Jalview.

Supplementary Table S1. Grouping and processing of duck Embryo and DF-1 cell samples for scientific research purposes.

Sample name	Sample incubation treatment	Sampling time
Embryo sample 1	200 µL of DTMUV Diluent	3 dpi
Embryo sample 2	200 µL of DTMUV Diluent	3 dpi
Embryo sample 3	200 µL of DTMUV Diluent	3 dpi
Embryo sample 4	200 µL of DTMUV Diluent	3 dpi
Embryo sample 5	200 µL of DTMUV Diluent	3 dpi
Embryo sample 6	200 µL of PBS buffer solution	3 dpi
Embryo sample 7	200 µL of PBS buffer solution	3 dpi
Embryo sample 8	200 µL of PBS buffer solution	3 dpi
Cell sample 1	10 µL of DTMUV Diluent	6 hpi
Cell sample 2	10 µL of DTMUV Diluent	12 hpi
Cell sample 3	10 µL of DTMUV Diluent	24 hpi
Cell sample 4	10 µL of DTMUV Diluent	36 hpi
Cell sample 5	10 µL of DTMUV Diluent	48 hpi
Cell sample 6	10 µL of DTMUV Diluent	60 hpi
Cell sample 7	10 µL of DTMUV Diluent	72 hpi
Cell sample 8	10 µL of DTMUV Diluent	48 hpi
Cell sample 9	10 µL of DTMUV Diluent	48 hpi
Cell sample 10	10 µL of 1640 medium	48 hpi
Cell sample 11	10 µL of 1640 medium	48 hpi

Supplementary Table S2. The sequences of five crRNAs designed by CRISPR-Offinder specifically targeting the *NS3* gene of DTMUV.

crRNA name	sequence (5’-3’)
NS3-crRNA-1	TTTGACTCAGAGTATCCTAAGTGC
NS3-crRNA-2	TTTGTGATAACCACTGACATCTCA
NS3-crRNA-3	TTTGGAGCGCAGCGGGTCATAGAT
NS3-crRNA-4	TTTGTGTCACATCCCTTCCAACCC
NS3-crRNA-5	TTTCGCTCATTGGAAAGAGGCCAA

Supplementary Table S3. Standard curve for 10-fold serial dilution and copy number quantification of the DTMUV *NS3* gene for 19 DTMUV-infected or uninfected samples.

Sample name	Copies/μL	CT(mean ± SEM)	C.V
NTC	/	39.32 ± 0.06	0.00
Standard curve 1	1.882×10^{10}	7.72 ± 0.64	0.14
Standard curve 2	1.882×10^{8}	13.53±0.20	0.02
Standard curve 3	1.882×10^{6}	20.09±0.01	0.00
Standard curve 4	1.882×10^{4}	27.59±0.05	0.00
Standard curve 5	1.882×10^{2}	35.42±1.05	0.05
Standard curve 6	1.882×10^{0}	38.46±0.69	0.03
Standard curve 7	1.882×10^{-1}	39.39±0.76	0.03
Embryo sample 1	4.280×10^{9}	10.07±0.20	0.03
Embryo sample 2	1.443×10^{6}	20.88±0.03	0.00
Embryo sample 3	6.229×10^{5}	22.16±0.02	0.00
Embryo sample 4	9.608×10^{8}	12.54±0.94	0.13
Embryo sample 5	1.297×10^{8}	13.04±1.52	0.20
Embryo sample 6	1.746×10^{1}	35.97±0.46	0.02
Embryo sample 7	4.116×10^{1}	35.70±0.21	0.01
Embryo sample 8	2.048×10^{1}	36.62±0.06	0.00
Cell Sample 1	5.006×10^{1}	35.42±0.69	0.03
Cell Sample 2	2.255×10^{4}	26.96±0.08	0.01
Cell Sample 3	3.949×10^{4}	25.83±0.08	0.01
Cell Sample 4	2.801×10^{6}	19.92±0.07	0.01
Cell Sample 5	1.126×10^{6}	21.21±0.05	0.00
Cell Sample 6	2.643×10^{6}	20.30±0.03	0.00
Cell Sample 7	8.882×10^{5}	21.70±0.04	0.00
Cell Sample 8	4.625×10^{6}	19.31±0.05	0.00
Cell Sample 9	1.893×10^{5}	23.82±0.06	0.00
Cell Sample 10	1.937×10^{1}	35.35±0.79	0.04
Cell Sample 11	1.338×10^{1}	36.50±0.46	0.02
